# The medial tangent of the proximal tibia is a suitable extra-articular landmark in determining the tibial anteroposterior axis

**DOI:** 10.1186/s12891-021-04206-8

**Published:** 2021-04-12

**Authors:** Hyunho Lee, Takanobu Sumino, Takashi Suzuki, Yutaka Sano, Noriyuki Endo, Yingshih Chang, Hirohisa Fujimaki, Keinosuke Ryu, Kazuyoshi Nakanishi

**Affiliations:** 1 Department of Orthopaedic Surgery, Nihon University School of Medicine, 30-1 Oyaguchi Kami-cho, Itabashi-ku, Tokyo, 173-8610 Japan; 2 Department of Orthopaedic Surgery, Eisei Hospital, 583-15 Kunugida, Hachioji-shi, Tokyo, 193-0942 Japan

**Keywords:** Tibia, Rotation, Reference, Total knee arthroplasty, Joint revision

## Abstract

**Background:**

Tibial rotational alignment in total knee arthroplasty (TKA) is generally determined based on intra-articular structure, and can be difficult to ascertain in some cases. The aim of this study was to investigate whether the medial tangent angle of the tibia (MTAT) could be useful in determining the anteroposterior axis of the tibia.

**Methods:**

This study was performed on 103 lower limbs in 53 patients who underwent primary total hip arthroplasty. The selection criteria for our study were based on the assumption that knees in patients undergoing THA exhibit fewer degenerative changes than knees in patients undergoing TKA. Using computed tomography images, the MTAT, comprising the medial tangent of the proximal tibia and the anteroposterior (AP) axis of the tibia, was measured on three horizontal planes: at the distal edge of the tibial tubercle (A), at 5 cm distally (B), and at 10 cm further distally (C). The tibial medial surface was grouped into three classes according to shape: valley type, flat type, and hill type. The percentage at which these shapes were observed in each group was also calculated. Measurement reliability was calculated using the intraclass correlation coefficient.

**Results:**

The angles were 45.2° (interquartile range: IR 43.0–47.7) at A, 42.7° (IR 38.7–45.9) at B, and 42.4° (IR 38.2–45.9) at C. Intra-rater reliability and inter-rater reliability was 0.982 and 0.974 at A, 0.810 and 0.411 at B, and 0.940 and 0.811 at C, respectively. Regarding the tibial medial surface, the valley type was observed in all cases at A, and the hill type was observed in the highest percentage of cases at B and C.

**Conclusions:**

The MTAT was approximately 45° at level A, and reproducibility was the highest among the three groups. The two points forming the valley on the tibial medial surface were bony ridges. Therefore, the medial tangent of the tibia at level A could be easily determined. Because the distal edge of the tibial tubercle exists at the surgical area and the extra-articular area, it can be a suitable intraoperative, extra-articular landmark in determining the tibial AP axis, even for revision TKA.

## Background

Total knee arthroplasty (TKA) is an effective procedure for painful knee osteoarthritis [1, 2]. However, it has been reported that 10 to 20% of patients who undergo TKA are dissatisfied with their surgical outcomes [3, 4]. Malrotation of the tibial component causes wear on the polyethylene tibial inserts which results in patellofemoral complications after TKA [5–8]. Therefore, tibial rotational alignment in TKA is an important factor for positive surgical outcomes. Rotational positioning of the tibial component in TKA is generally determined based on intra-articular structure [9–13]. However, this determination is often difficult in cases with severe deformity or inflammation, in which there are osteophytes or degenerative changes in the posterior cruciate ligament (PCL). Moreover, in revision TKA the intra-articular structure has already been resected. Consequently, other extra-articular landmarks for determining tibial rotational alignment are required for such cases. However, the potential extra-articular landmarks investigated to date have been reported to be unreliable [14].

The medial surface of the tibia is flat and smooth [15], and the cortex is often used for bone graft [16]. Therefore, it was hypothesized that the cortex surface could be used as a landmark for tibial rotational alignment. This area is an extra-articular area, and can be used as a reference even in revision TKA. The aim of this study was to investigate whether the medial tangent angle of the tibia (MTAT), comprising the medial tangent of the proximal tibia and the anteroposterior (AP) axis of the tibia, could be useful in determining tibial rotational alignment in TKA in general, and revision TKA in particular.

## Methods

### Study population

A cross sectional study was performed on 103 lower limbs in 53 Japanese patients scheduled to undergo primary total hip arthroplasty (THA) at our hospital from April, 2018 to March, 2019 (70 lower limbs in 36 female patients and 33 lower limbs in 17 male patients). The selection criteria for our study were based on the assumption that knees in patients undergoing THA exhibit fewer degenerative changes than knees in patients undergoing TKA. In addition, computed tomography (CT) data were obtained from all subjects during routine preoperative planning for the hip arthroplasty. Patients with rheumatoid arthritis, patients who had a history of previous surgery in the lower limbs, and patients with osteoarthritis (OA) of the knee equal to or higher than Kellgren-Lawrence classification grade 2 were excluded [17], because measurement of the MTAT was likely to be affected by the deformities inherent in such knees. The median age was 65.0 years (interquartile range: IR 57.0–71.0 years), the median body height was 157.2 cm (IR 150.7–164.3 cm), the median body weight was 55.0 kg (IR 50.9–62.0 kg), the median body mass index was 22.3 kg/m^2^ (IR 20.5–24.3 kg/m^2^), and the median femorotibial angle was 176.0 ° (IR 174.0–178.0 °).

The Institutional Review Board (IRB) of Nihon University School of Medicine granted ethical approval (approval number: RK-200714-8). All patients of our study gave their informed consent for participation and publication of their anonymized data. All procedures performed in studies involving human participants were in accordance with the 1964 Helsinki declaration.

### Measurement of the angle between the medial tangent of the proximal tibia and the anteroposterior axis of the tibia using computed tomography images

**Fig. 1:**
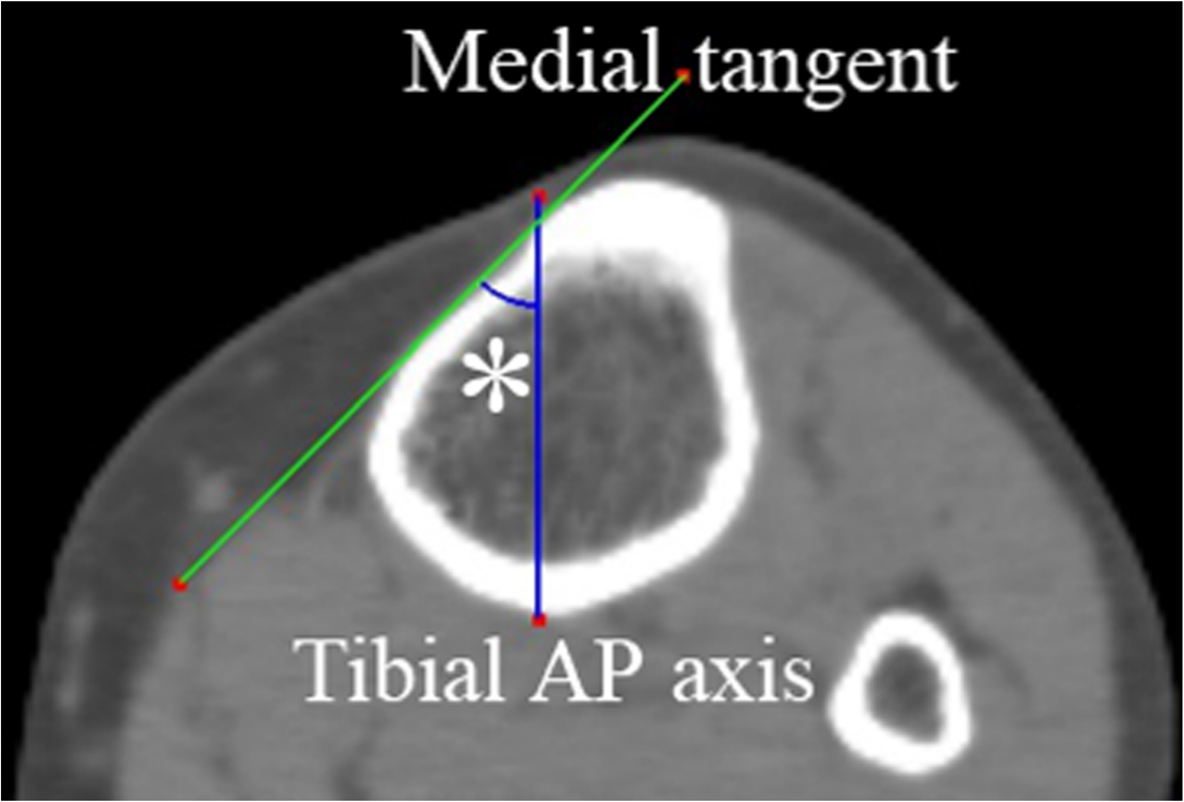
The medial tangent angle of the tibia on the horizontal plane. AP, anteroposterior. An asterisk indicates the medial tangent angle of the tibia (MTAT), comprising the medial tangent of the proximal tibia and the anteroposterior axis of the tibia

**Fig. 2:**
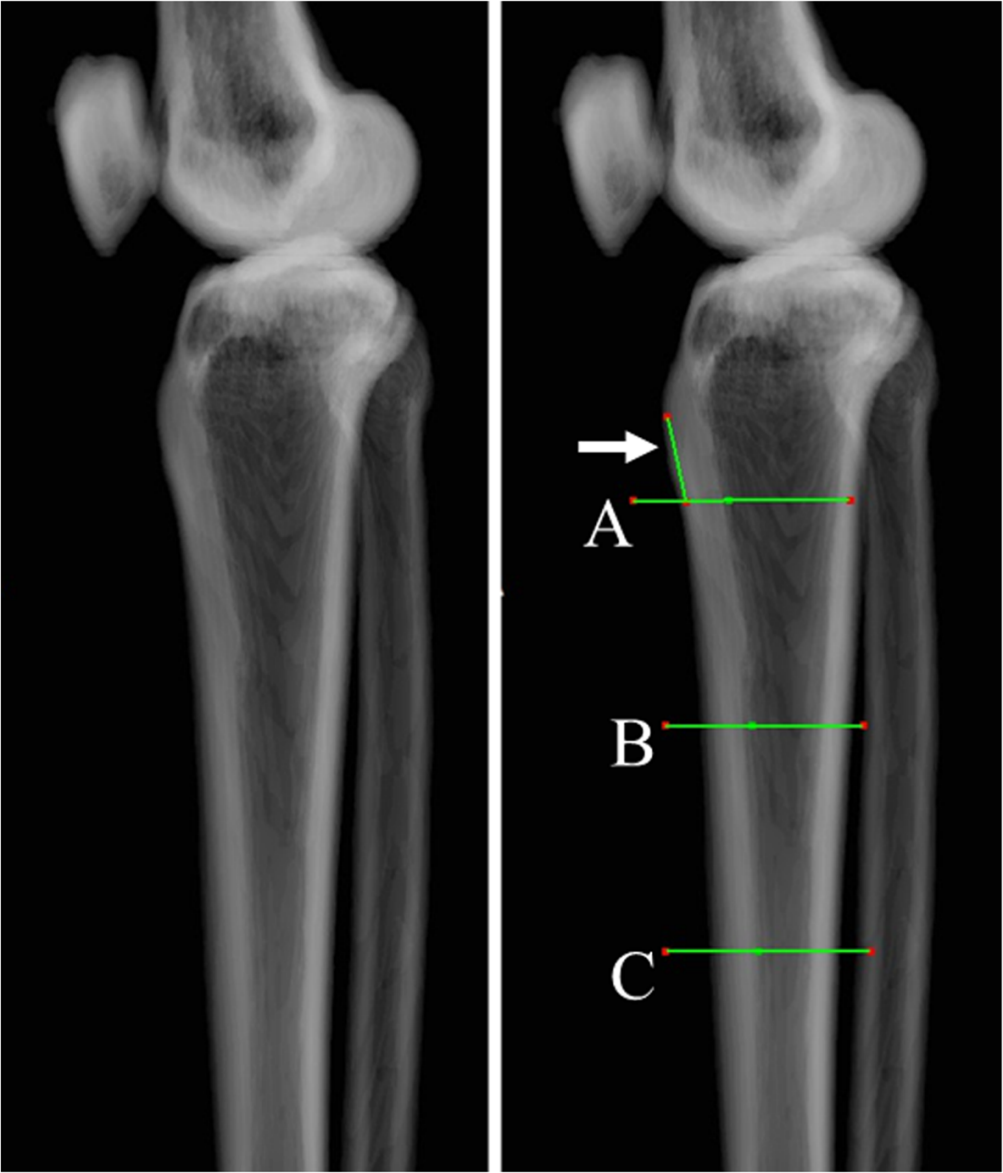
Levels of the three horizontal planes in measuring the medial tangent angles of the tibia. **a** the level of the distal edge of the tibial tubercle; **b** the level at 5 cm distal to (**a**); **c** the level at 10 cm further distally. The distal edge of the tibial tubercle was defined as the inflection point on the downslope of the tibial tubercle. A white arrow indicates the distance between the top of the tibia tubercle and the distal edge of the tibial tubercle

**Fig. 3:**
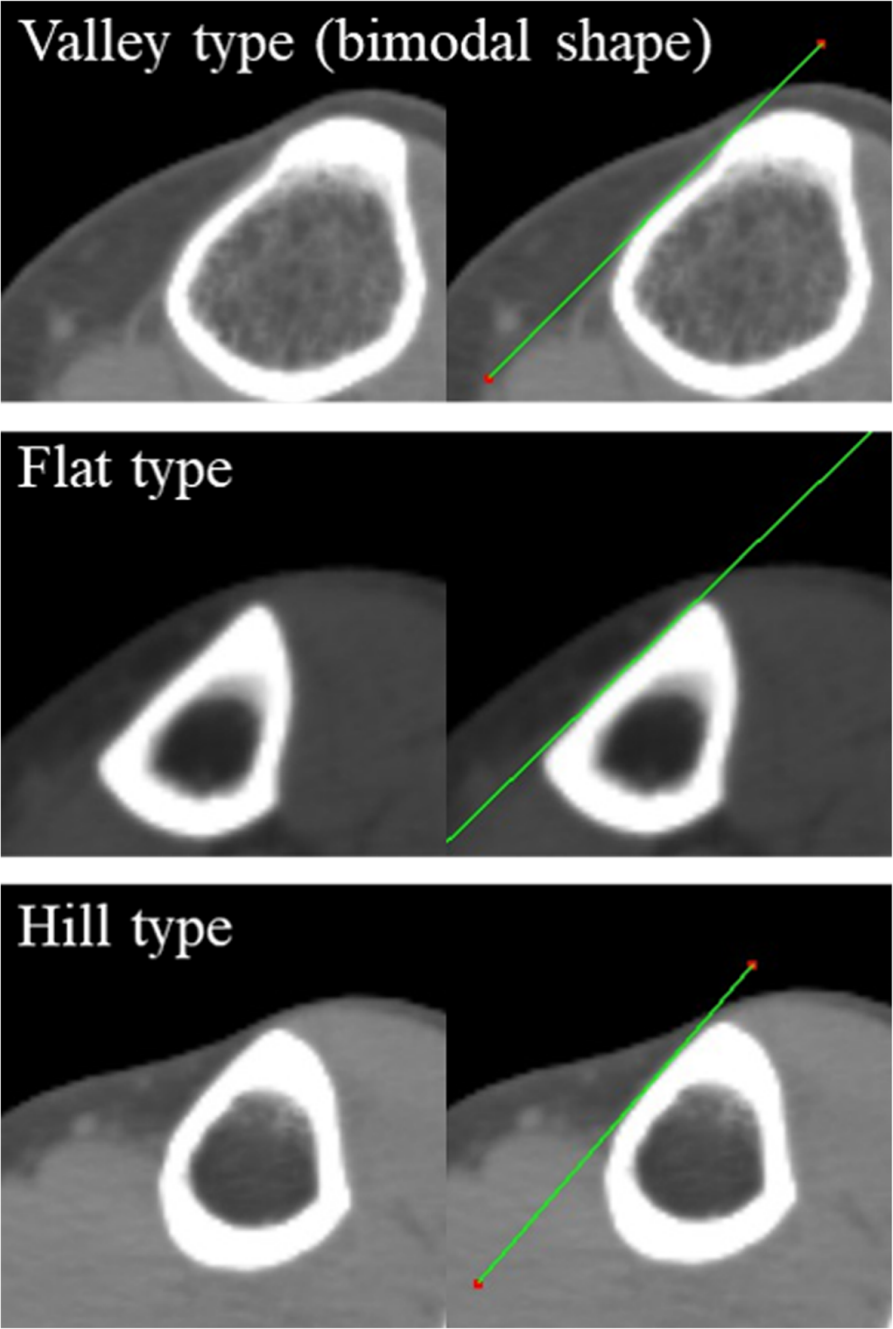
Classification according to shape of the medial surface on the horizontal plane. The tibial medial surface was grouped into three classes according to shape: valley type (bimodal shape), flat type, and hill type

The tibia model was created using three-dimensional template software (ZedKnee®^□^, LEXI, Japan). Coordinate axes were as follows. Origin: the intersection point between the Z-axis and the perpendicular line running down from the middle of the PCL to the Z-axis, Z-axis: the line passing through the center point of the medullary cavity at the proximal one third and the center point at the distal one third, Y-axis: the line that is perpendicular to the Z-axis and passing through the origin, and is on the plane formed by the Z-axis and the line segment parallel to the tibial AP axis and intersecting the Z- axis, X-axis: the line passing through the origin and perpendicular to both the Z-axis and the Y-axis. The tibial AP axis was defined as the line connecting the middle of the PCL and the medial edge of the patellar tendon attachment [11, 18]. The MTAT, comprising the medial tangent of the proximal tibia and the anteroposterior (AP) axis of the tibia, was measured on three horizontal planes (X-Y planes): at the distal edge of the tibial tubercle (A), at 5 cm distally (B), and at 10 cm further distally (C) (Figs. 1, 2). The distal edge of the tibial tubercle was defined as the inflection point on the downslope of the tibial tubercle. Differences in the MTAT value between the three levels, between the right and left lower limbs, and between females and males were calculated. The tibial medial surface was grouped into three classes according to shape: valley type (bimodal shape), flat type, and hill type (Fig. 3). The percentage at which these shapes were observed in each group (A, B and C) was also calculated. In addition, the distance between the top of the tibia tubercle and the distal edge of the tibial tubercle was also measured (Fig. 2).

### Statistical analysis

Data analysis was performed using IBM SPSS software, version 25. The Friedman’s test was used to investigate the differences in the MTAT value between the three groups (A, B and C). The Wilcoxon’s signed rank test was performed to investigate the differences between the right and left lower limbs. The Wilcoxon’s rank sum test was performed to investigate the difference between females and males. The Fisher’s exact test was used to investigate the differences in the percentage at which the three shapes were observed between the three groups (A, B and C). *P* values of less than 0.05 were considered significant. Intra-rater and inter-rater reliability for measuring the MTAT at the three horizontal planes was calculated with the intraclass correlation coefficient. The MTAT at each of the three horizontal planes was measured two times with an interval of 6 weeks by two investigators on ten knees randomly selected from the study subjects.

## Results

The median MTAT was 45.2° (IR 43.0–47.7) at A, 42.7° (IR 38.7–45.9) at B, and 42.4° (IR 38.2–45.9) at C (Table 1). Significant differences were noted between the values at A and B, and between the values at A and C. Intra-rater reliability and inter-rater reliability for measuring the MTAT was 0.982 and 0.974 at A, 0.810 and 0.411 at B and 0.940 and 0.811 at C, respectively (Table 1). No significant differences between right and left lower limbs or between females and males were noted (Tables 2 and 3). Regarding the tibial medial surface, the valley type was observed in all cases at A, and the hill type was observed in the highest percentage of cases at B and C (Table 4). Significant differences in the percentage at which the three shapes were observed were noted between group A and B, and between group A and C. The median distance between the top of the tibia tubercle and the distal edge of the tibial tubercle was 17.8 mm (IR 15.7–19.8 mm).

**Table 1:** Measurement of the medial tangent angle of the tibia and reliability calculated with intraclass correlation coefficients

	Level	Median	interquartile range	Inter-rater reliability	Intra-rater reliability
Whole (*n* = 103)	A	45.2^***, †††^	43.0–47.7	0.974	0.982
	B	42.7^***^	38.7–45.9	0.411	0.810
	C	42.4^†††^	38.2–45.9	0.811	0.940

A, the level of the distal edge of the tibial tubercle; B, the level at 5 cm distal to A; C, the level at 10 cm further distally

Data are presented as degrees, intraclass correlation coefficients

An asterisk indicates significant difference between the values at A and B. ^***^P < 0.001. A dagger indicates significant difference between the values at A and C. ^†††^*P* < 0.001

**Table 2:** Measurement of the medial tangent angle of the tibia in the right and left lower limbs

	Level	Median	interquartile range	*P* value
Right (*n* = 50) / Left (*n* = 50)	A	45.3/45.3	42.2–48.0/43.0–47.2	0.224
	B	43.0/42.0	38.7–46.0/39.1–45.4	0.772
	C	42.9/42.1	39.0–46.0/37.9–45.2	0.069

A, the level of the distal edge of the tibial tubercle; B, the level at 5 cm distal to A; C, the level at 10 cm further distally

Data are presented as degrees or *P* values

**Table 3:** Measurement of the medial tangent angle of the tibia in females and males

	Level	Median	interquartile range	*P* value
Females (*n* = 70) / Males (*n* = 33)	A	45.3/45.1	43.0–48.0/42.9–46.9	0.611
	B	41.1/43.5	38.3–45.4/40.7–45.9	0.159
	C	42.8/42.1	38.9–46.0/37.5–45.4	0.360

A, the level of the distal edge of the tibial tubercle; B, the level at 5 cm distal to A; C, the level at 10 cm further distally

Data are presented as degrees or *P* values

**Table 4:** Percentage of cases exhibiting each tibial medial surface shape at the three levels

	Valley type	Flat type	Hill type
A (*n* = 103)	100%	0%	0%
B (*n* = 103)	5.8%	17.5%	76.7%
C (*n* = 103)	13.6%	20.4%	66.0%

Significant differences in the percentage at which the three shapes were observed were noted between group A and B (*P* value < 0.001), and between group A and C (*P* value < 0.001)

A, the level of the distal edge of the tibial tubercle; B, the level at 5 cm distal to A; C, the level at 10 cm further distally

Data are presented as percentages

## Discussion

The most important finding of the present study was that the medial tangent of the proximal tibia was a suitable extra-articular landmark in determining the tibial anteroposterior axis for TKA, and the MTAT was approximately 45° at level A.

In the past, tibial rotational alignment in TKA had been determined in reference to the medial one third of the tibial tubercle [12]. However, there was no strong theoretical justification for this method. Akagi et al. demonstrated that the line connecting the middle of the PCL to the medial edge of the patellar tendon attachment was perpendicular to the surgical epicondylar axis, and this line has since been used as the tibial AP axis for primary TKA [11, 18, 19]. Since then, variants of this line have been reported [20, 21]. Kim et al. reported that the line connecting the anterior border of the proximal third of the tibia to the middle of the PCL was also perpendicular to the surgical epicondylar axis, and could also be used as the tibial AP axis [22]. These lines are very useful for primary TKA in which the PCL is recognized directly. For primary medial unicompartmental knee arthroplasty, Tsukamoto et al. showed that the line connecting the medial border of the patellar tendon at the articular surface level and the medial intercondylar tubercle was suitable as the tibial AP axis [23]. However, the above lines cannot be used for knees in which the PCL disappears due to severe inflammation or for revision TKA in which joint structures have already been resected. Additional landmarks for tibial rotational alignment independent of intra-articular condition are needed for such cases.

In techniques referencing the extra-articular structure, the transmalleolar axis of the ankle and the second metatarsus bone axis of the foot have been used to determine the tibial AP axis conventionally [12]. However, it has been reported that these techniques exhibit individual variability [14, 24, 25]. Though reproducibility of the MTAT value at level C in the present study was high, the interquartile range of the MTAT at level C was the largest among the three groups (Table 1). Individual variability of the tibial torsion was suggested even in the present study. This shows that extra-articular reference in the determination of the tibial AP axis should be close to the knee. Therefore, the medial tangent of the proximal tibia was determined to be a suitable landmark. However, it was also considered that the MTAT at a level more proximal than level A would also vary between individuals because of the individual variability that exists in the protrusion of the tibial tubercle. In addition, medial bony defect or spur was often observed at levels more proximal than level A. Therefore, the MTAT was evaluated at three levels (A, B and C) in the present study. The median distance between the top of the tibia tubercle and the distal edge of the tibial tubercle was 17.8 mm (IR 15.7–19.8 mm), or about one finger in the present study. Therefore, if it is difficult to identify the distal edge of the tibial tubercle, the MTAT at a level one finger distal to the top of the tibia tubercle may be used as a reference.

Even when the identification of the lines illustrated above is easy, it is difficult to confirm whether such lines do in fact match the preoperatively planned tibial axis of rotation. Mitsuhashi et al. showed that the number of outliers for rotational alignment of the tibial component was significantly higher in conventional TKA than in TKA using a navigation system [26]. However, because TKA using a navigation system involves higher costs, there are some hospitals at which such systems are not used. In addition, the tracker pins in such systems have been known to cause infection and fracture, although incidence rates are low [27, 28]. Therefore, intraoperative landmarks not involving a navigation system are more practical for the determination of tibial rotational alignment.

**Fig. 4:**
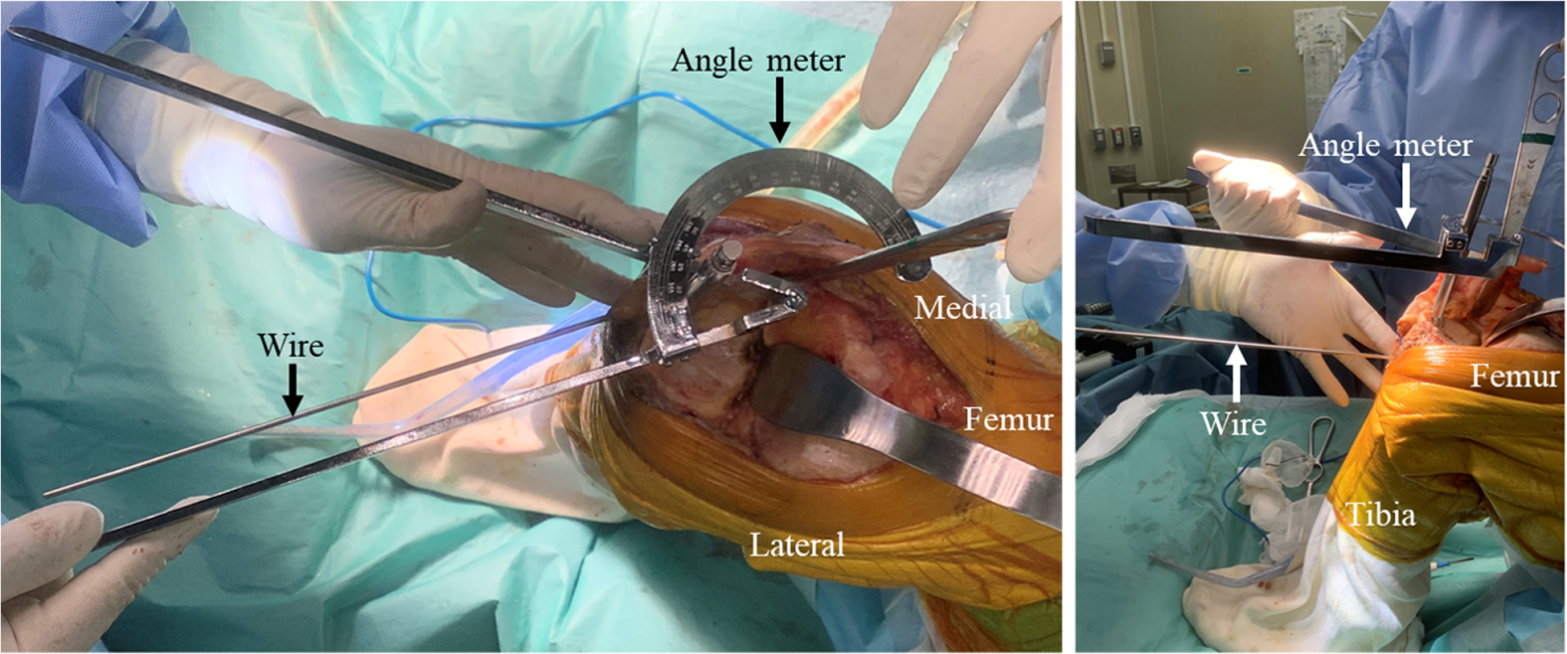
Intraoperative reference for the tibial anteroposterior axis with the medial tangent angle of the tibia. The medial tangent of the tibia can be reproduced by inserting a wire along the medial surface of the tibia. Intraoperative confirmation of the anteroposterior axis of the tibia can be performed with the medial tangent angle of the tibia

The distal edge of the tibial tubercle exists at, or very close to, the surgical area. The main medial anatomical structure on the horizontal plane at level A is the just inferior portion of the pes anserinus, which inserts into the tibia tubercle 6 ± 5 mm distally [29, 30]. Therefore, by inserting a wire along the surface of the cortex through the thin portion of the pes anserinus, reproduction of the medial tangent of the tibia during surgery can be achieved (Fig. 4). In addition, because the MTAT at level A was found to be approximately 45° in the present study, the value can be easily used as an intraoperative reference for the tibial AP axis. Even in conventional TKA not involving a navigation system, the MTAT at level A can be a very useful landmark.

The medial surface of the tibia was smooth, but not completely flat (Table 4). In all lower limbs, the tibial medial surface at level A was found to be of the valley type due to the fact that the distal edge of the tibial tubercle is still slightly prominent at this level (Table 4). In addition, the two points forming the valley were bony ridges. Therefore, the medial tangent of the tibia at level A could be easily determined. As a result, the reproducibility of the MTAT value at level A was the highest among the three groups (Table 1).

There are some limitations to the present study. First, individual variability existed, even at level A. However, because the interquartile range of the MTAT at level A in our study was small, we believe that a value of 45° may be used by surgeons intraoperatively. Surgeons may also choose to reproduce the accurate MTAT intraoperatively, using the value of the MTAT at level A measured on preoperative CT scans. In revision cases in which the MTAT cannot be measured in preoperative CT, the MTAT on the contralateral side may be used as a reference since no significant difference between the right and left lower limbs was noted in our study (Table 2). Second, the patients in the present study were patients scheduled to undergo total hip arthroplasty. However, some patients with dysplasia of the hip have been reported to have lower limb rotational alignment abnormalities [31]. Therefore, although patients with moderate to severe OA in the knee were excluded due to the fact that degenerative changes were considered likely to affect the identification of the tibial AP axis on CT images, future studies should focus on patients scheduled to undergo knee arthroplasty. Furthermore, since the aim of our study was to investigate the MTAT using CT images, we deemed it unjustifiable to expose healthy subjects to unnecessary radiation exposure, and selected patients already scheduled to undergo lower limb CT scans. Third, the patients in the present study were all Japanese. Although the median MTAT at level A was approximately 45° in the present study, it is possible that different bony shapes in the lower limbs exist in different ethnicities. In the future more research involving subjects with osteoarthritic knee and in other ethnic groups should be conducted.

## Conclusions

The MTAT was approximately 45° at the distal edge of the tibial tubercle with high reproducibility. In addition, because this level exists at the surgical area and the extra-articular area, it can be a suitable intraoperative, extra-articular landmark in determining the tibial AP axis, even for revision TKA, in which the PCL has already been resected.

## Data Availability

The datasets generated and/or analyzed during the current study are not publicly available due to our institutional policy but are available from the corresponding author on reasonable request.

## Abbreviations

AP: Anteroposterior
CT: Computed tomography
IR: Interquartile range
IRB: Institutional review board
MTAT: Medial tangent angle of the tibia
OA: Osteoarthritis
PCL: Posterior cruciate ligament
THA: Total hip arthroplasty
TKA: Total knee arthroplasty

## References

[1] Shan L, Shan B, Suzuki A, Nouh F, Saxena A (2015). Intermediate and long-term quality of life after total knee replacement: a systematic review and meta-analysis. J Bone Joint Surg Am.

[2] Anderson JG, Wixson RL, Tsai D, Stulberg SD, Chang RW (1996). Functional outcome and patient satisfaction in total knee patients over the age of 75. J Arthroplast.

[3] Noble PC, Conditt MA, Cook KF, Mathis KB (2006). The John Insall award: patient expectations affect satisfaction with total knee arthroplasty. Clin Orthop Relat Res.

[4] Bourne RB, Chesworth BM, Davis AM, Mahomed NN, Charron KD (2010). Patient satisfaction after total knee arthroplasty: who is satisfied and who is not?. Clin Orthop Relat Res.

[5] Wasielewski RC, Galante JO, Leighty RM, Natarajan RN, Rosenberg AG (1994). Wear patterns on retrieved polyethylene tibial inserts and their relationship to technical considerations during total knee arthroplasty. Clin Orthop Relat Res.

[6] Berger RA, Crossett LS, Jacobs JJ, Rubash HE (1998). Malrotation causing patellofemoral complications after total knee arthroplasty. Clin Orthop Relat Res.

[7] Panni AS, Ascione F, Rossini M, Braile A, Corona K, Vasso M, Hirschmann MT (2018). Tibial internal rotation negatively affects clinical outcomes in total knee arthroplasty: a systematic review. Knee Surg Sports Traumatol Arthrosc.

[8] Steinbrück A, Schröder C, Woiczinski M, Müller T, Müller PE, Jansson V, Fottner A (2016). Influence of tibial rotation in total knee arthroplasty on knee kinematics and retropatellar pressure: an in vitro study. Knee Surg Sports Traumatol Arthrosc.

[9] Uehara K, Kadoya Y, Kobayashi A, Ohashi H, Yamano Y (2002). Bone anatomy and rotational alignment in total knee arthroplasty. Clin Orthop Relat Res.

[10] Dalury DF (2001). Observations of the proximal tibia in total knee arthroplasty. Clin Orthop Relat Res.

[11] Akagi M, Oh M, Nonaka T, Tsujimoto H, Asano T, Hamanishi C (2004). An anteroposterior axis of the tibia for total knee arthroplasty. Clin Orthop Relat Res.

[12] Insall JN, Easley ME, Insall JN, Scott WN (2001). Surgical techniques and instrumentation in total knee arthroplasty. Surgery of the knee. 2.

[13] Cobb JP, Dixon H, Dandachli W, Iranpour F (2008). The anatomical tibial axis: reliable rotational orientation in knee replacement. J Bone Joint Surg Br.

[14] Akagi M, Mori S, Nishimura S, Nishimura A, Asano T, Hamanishi C (2005). Variability of extraarticular tibial rotation references for total knee arthroplasty. Clin Orthop Relat Res.

[15] Wasielewski RC, Callaghan JJ, Rosenberg AG, Rubash HE, Simonian PT, Wickiewicz TL (2003). Surgical anatomy of the knee. The adult knee.

[16] Crenshaw AH, Azar FM, Beaty JH, Canale ST (2017). Surgical techniques and approaches. Campbell's operative orthopaedics 1.

[17] Kellgren JH, Lawrence JS (1957). Radiological assessment of osteo-arthrosis. Ann Rheum Dis.

[18] Aglietti P, Sensi L, Cuomo P, Ciardullo A (2008). Rotational position of femoral and tibial components in TKA using the femoral transepicondylar axis. Clin Orthop Relat Res.

[19] Shukla S, Upadhyaya V, Goel M, Gupta S (2019). Antero-posterior axis of tibia in patient undergoing total knee replacement in Indian population. J Clin Orthop Trauma.

[20] Drexler M, Backstein D, Studler U, Lakstein D, Haviv B, Schwarzkopf R, Rutenberg TF, Warschawski Y, Rath E, Kosashvili Y (2017). The medial border of the tibial tuberosity as an auxiliary tool for tibial component rotational alignment during total knee arthroplasty (TKA). Knee Surg Sports Traumatol Arthrosc.

[21] Sahin N, Atici T, Kurtoglu U, Turgut A, Ozkaya G, Ozkan Y (2013). Centre of the posterior cruciate ligament and the sulcus between tubercle spines are reliable landmarks for tibial component placement. Knee Surg Sports Traumatol Arthrosc.

[22] Kim CW, Seo SS, Kim JH, Roh SM, Lee CR (2014). The anteroposterior axis of the tibia in Korean patients undergoing total knee replacement. Bone Joint J.

[23] Tsukamoto I, Akagi M, Mori S, Inoue S, Asada S, Matsumura F (2017). Anteroposterior rotational references of the tibia for medial Unicompartmental knee Arthroplasty in Japanese patients. J Arthroplast.

[24] Yagi T (1994). Tibial torsion in patients with medial-type osteoarthrotic knees. Clin Orthop Relat Res.

[25] Yoshioka Y, Siu DW, Scudamore RA, Cooke TD (1989). Tibial anatomy and functional axes. J Orthop Res.

[26] Mitsuhashi S, Akamatsu Y, Kobayashi H, Kusayama Y, Kumagai K, Saito T (2018). Combined CT-based and image-free navigation systems in TKA reduces postoperative outliers of rotational alignment of the tibial component. Arch Orthop Trauma Surg.

[27] Beldame J, Boisrenoult P, Beaufils P (2010). Pin track induced fractures around computer-assisted TKA. Orthop Traumatol Surg Res.

[28] Kamara E, Berliner ZP, Hepinstall MS, Cooper HJ (2017). Pin site complications associated with computer-assisted navigation in hip and knee Arthroplasty. J Arthroplast.

[29] Reina N, Abbo O, Gomez-Brouchet A, Chiron P, Moscovici J, Laffosse JM (2013). Anatomy of the bands of the hamstring tendon: how can we improve harvest quality?. Knee..

[30] Ivey M, Prud'homme J (1993). Anatomic variations of the pes anserinus: a cadaver study. Orthopedics..

[31] Lerch TD, Liechti EF, Todorski IAS, Schmaranzer F, Steppacher SD, Siebenrock KA (2020). Prevalence of combined abnormalities of tibial and femoral torsion in patients with symptomatic hip dysplasia and femoroacetabular impingement. Bone Joint J.

